# Predictors of Non-Adherence to Breast Cancer Screening among Hospitalized Women

**DOI:** 10.1371/journal.pone.0145492

**Published:** 2015-12-28

**Authors:** Waseem Khaliq, Ali Aamar, Scott M. Wright

**Affiliations:** 1 Department of Medicine, Johns Hopkins Bayview Medical Center, Johns Hopkins University, School of Medicine, Baltimore, Maryland, United States of America; 2 Department of Internal Medicine, Yale-Waterbury Hospital, Yale School of Medicine, Waterbury, Connecticut, United States of America; Wayne State University School of Medicine, UNITED STATES

## Abstract

**Objective:**

Disparities in screening mammography use persists among low income women, even those who are insured, despite the proven mortality benefit. A recent study reported that more than a third of hospitalized women were non-adherent with breast cancer screening. The current study explores prevalence of socio-demographic and clinical variables associated with non-adherence to screening mammography recommendations among hospitalized women.

**Patients and Methods:**

A cross sectional bedside survey was conducted to collect socio-demographic and clinical comorbidity data thought to effect breast cancer screening adherence of hospitalized women aged 50–75 years. Logistic regression models were used to assess the association between these factors and non-adherence to screening mammography.

**Results:**

Of 250 enrolled women, 61% were of low income, and 42% reported non-adherence to screening guidelines. After adjustment for socio-demographic and clinical predictors, three variables were found to be independently associated with non-adherence to breast cancer screening: low income (OR = 3.81, 95%CI; 1.84–7.89), current or ex-smoker (OR = 2.29, 95%CI; 1.12–4.67), and history of stroke (OR = 2.83, 95%CI; 1.21–6.60). By contrast, hospitalized women with diabetes were more likely to be compliant with breast cancer screening (OR = 2.70, 95%CI 1.35–5.34).

**Conclusion:**

Because hospitalization creates the scenario wherein patients are in close proximity to healthcare resources, at a time when they may be reflecting upon their health status, strategies could be employed to counsel, educate, and motivate these patients towards health maintenance. Capitalizing on this opportunity would involve offering screening during hospitalization for those who are overdue, particularly for those who are at higher risk of disease.

## Introduction

Breast cancer is the most common malignancy and the second leading cause of cancer death among women in the United States [1,2]. Mammography remains the screening test of choice and has been shown to reduce breast cancer mortality by 22–35% among women older than 50 year [3,4]. Studies evaluating barriers to breast cancer screening have reported that mammography utilization rates are associated with socioeconomic status (SES), level of educational, access to a health care, and race [5–8]. Such associations have been established in the general population, or community-based cohorts since screening mammography testing has been exclusively promoted in the outpatient clinics [5–8].

Efforts are needed to increase early detection of breast cancer especially among the groups of women who are of lower socioeconomic status, and with limited access to healthcare. In this vulnerable group, low screening rates translate into postponement in early breast cancer detection and poor prognosis [9]. Patients’ preferences for cancer screening are also known to significantly influence utilization, and thus impact health outcomes [10]. In a study of hospitalized women evaluating breast cancer screening trends, approximately forty percent of the women age 50–75 years were found to be non-adherent to breast cancer screening despite the fact that 95% of the women had healthcare insurance [11]. For these women, the lack of transportation and forgetting screening appointments were reported as major barriers to compliance [11]. Interestingly, a majority (76%) expressed preferences for having inpatient screening mammogram while they were in the hospital.

Hospitalized patients can be thought of as a distinct subset of general population. By a function of this unifying feature, they are less well than others. During a hospitalization, they are a “captive audience”, and at this time they may be reflecting on their overall health [12]. Overtime, hospitalized patients represent a substantial subset of the larger population. With responsibility for maintaining wellness and averting sickness, some forward thinking healthcare delivery entities (e.g. Kaiser Permanente) and Accountable Care Organizations are beginning prioritize offering screening whenever and wherever patients interface with the delivery system [13].

The purpose of study was to explore and quantify the socio-demographic and clinical variables associated with non-adherence to breast cancer screening among hospitalized women. We hypothesized that a combination of socio-demographic variables and comorbidities would best explain the association of non-adherence with breast cancer screening.

## Methods

### Study Design and Sample

Detailed enrollment methods have been published [11]. Briefly, four hundred and twenty seven hospitalized women met eligibility criteria during the study period. Of them, 59 (14%) refused to participate, 47 (11%) were excluded due to history of breast cancer, and 71 (17%) women were discharged from the hospital before the study coordinator could consent them. This left a study population with 250 women.

### Data Collection and Measures

Data was collected by a research assistant via bedside interviews that lasted approximately 10 minutes. The survey included questions regarding socio-demographic information such as race, education, and annual household income. Low socioeconomic status (SES) was defined as self-reported total annual household income less than $20,000; this was selected instead of poverty level to avoid the necessity of knowing family size and number of dependents. Nine patients elected not to report annual household income. ‘Non-adherence with breast cancer screening’ recommendations was defined as having had a screening mammography more than 24 months before participating in the study survey among women age 52 or older—in accordance with guidelines by the United States Preventive Services Task Force (USPSTF) [14]. Access to health care was determined by determining health insurance status, and whether patients had a primary care doctor. Disease burden was evaluated by probing about several medical comorbidities including coronary artery disease, hypertension, hyperlipidemia, atrial fibrillation, chronic kidney disease, thromboembolism, obstructive sleep apnea, osteoporosis, depression, hypothyroidism, nephrolithiasis, chronic hepatitis, anemia, and cancers other than skin or breast. Family history of breast cancer was judged to be positive in subjects reporting a breast cancer diagnosis in first-degree relatives (namely mother, sisters, or daughters). Several questions regarding reproductive history were asked to generate the ‘*Gail Risk Prediction Score’—*probability of developing breast cancer within the next 5 years according to the National Cancer Institute Breast Cancer Risk Tool (http://www.cancer.gov/bcrisktool/) [15–20].

Pilot testing of the survey was conducted on fifteen patients to enhance question clarity and reduce ambiguity. The Institutional Review Board at Johns Hopkins Bayview Medical Center approved the pilot survey and the study protocol. The study participants provide their written informed consent for study participation.

### Statistical Analysis

Respondent characteristics are presented as proportions and means. Unpaired *t* test and Chi square tests were used to compare demographic and socioeconomic characteristics based on adherence to breast cancer screening. We used Logistic regression models for analyses to predict the odds of non-adherence with breast cancer screening among the hospitalized women. Logistic regression models were used for both socio-demographic models and medical comorbidity burden models separately, and the resultant models were then combined. The survey data were analyzed in March 2015 using Stata statistical software (StataCorp LP, Version 12.1).

## Results

The mean age of the study population was 61.5 years, 31% were African American, and 6% were uninsured. Sixty one percent women reported an annual household income less than $20,000. One third of the study population (32%) was at high risk for breast cancer based on 5-year-risk prediction using the Gail model (Gail score ≥ 1.7%). Forty two percent of the women studied were ‘non-adherent’ or overdue for breast cancer screening, of whom 16% had never had a mammogram. Characteristics of the study participants are shown in Table 1, grouped according to their breast cancer screening adherence status. While most variables were similar across the two groups, non-adherent women were more likely to be of low income, medically uninsured, smokers, and lacking a primary care provider.

**Table 1:** Study population characteristics.

Characteristics	All participants (N = 250)	Adherent (N = 146)	Non-adherent (N = 104)	p value
**Age > 60 years, n (%)**	126 (50)	76 (57)	50 (54)	0.54
**Race**				
**Caucasian, n (%)**	164 (66)	94 (64)	70 (67)	0.82
**African American, n (%)**	77 (31)	46 (32)	31 (30)	
**Others, n (%)**	9 (3)	6 (4)	3 (3)	
**Education less than high school, n (%)**	82 (33)	42 (28)	40 (38)	0.11
**Medically uninsured, n (%)**	15 (6)	5 (3)	10 (10)	0.04
**No primary care physician, n (%)**	23 (9)	7 (5)	16 (15)	0.004
**BMI kg/m** ^**2**^ **, mean (SD)**	33.8 (11)	33.4 (10.7)	34.5 (11.5)	0.45
**BMI kg/m** ^**2**^ **as categories**				
**Reference 16.9–24.99, n (%)**	50 (20)	33 (23)	17 (16)	
**Pre-obese 25–29.99, n (%)**	58 (23)	32 (22)	26 (25)	
**Obese ≥ 30, n (%)**	142 (57)	81 (55)	61 (59)	
**Chronic disability or wheel chair bound patients, n (%)**	105 (42)	59 (40)	46 (44)	0.55
**Family History of breast cancer, n (%)**	34 (14)	18 (12)	16 (15)	0.48
**Annual income less than $20,000, n (%)**	148 (61)	74 (52)	74 (71)	0.000
**5 year GAIL Score ≥ 1.7, n (%)**	81 (32)	52 (36)	29 (28)	0.2
**3 or more comorbidities, n (%)**	187 (75)	113 (60)	74 (40)	0.26
**Current/ever smoker, n (%)**	73 (29)	34 (23)	39 (38)	0.02

In unadjusted, bivariate analysis, 2 of the 9 socio-demographic variables and 1 of the 14 clinical variables were associated with non-adherence with breast cancer screening recommendations (Table 2). Logistic regression analysis (Table 2) showed that odds of non-adherence to breast cancer screening recommendations were more than 3 times higher (OR: 3.56; 95% CI 1.79–7.07) among low income women as compared to those with higher income, and almost double (OR: 1.99, CI 1.06–3.75) for current or ex-smoker compared to never smoker hospitalized women after adjustment for all socio-demographic variables. Similarly, in the multivariable regression analyses of clinical variables only, women with history of cerebrovascular accidents were more than twice as likely (OR: 2.73, 95% CI 1.27–5.89) to be non-adherent to breast cancer screening. Women with a history of diabetes were far more likely to be adherent to breast cancer screening as compared to women without diabetes (OR 0.49, 95%CI 0.26–0.90).

**Table 2:** Unadjusted and adjusted logistic regression analyses for associations with non-adherence to breast cancer screening recommendations among hospitalized women.

Suspected Non-adherence Risk factors	Odds Ratio (95% CI)			
	Unadjusted	Adjusted Model 1^a^	Adjusted Model 2^b^	Adjusted Model 3^c^
***Social and demographic factors***				
**Age > 60 years**	0.85 (0.52–1.41)	1.05 (0.59–1.89)	–	0.90 (0.46–1.74)
**African American and other races (versus Caucasians)**	0.88 (0.52–1.49)	1.08 (0.59–1.97)	–	1.1 (0.56–2.12)
**Married**	1.01 (0.59–1.72)	0.58 (0.30–1.14)	–	0.61 (0.39–1.24)
**Less than a high school education**	1.55 (0.91–2.64)	1.23 (0.66–2.29)	–	1.46 (0.75–2.84)
**Employed**	1.02 (0.54–1.91)	0.60 (0.28–1.29)	–	0.55 (0.23–1.29)
**Medically uninsured**	3.00 (1.00–9.10)	1.82 (0.50–6.63)	–	1.77 (0.46–6.87)
**Annual household income less than $20,000**	**2.87 (1.63–5.06)**	**3.56 (1.79–7.07)**	–	**3.81 (1.84–7.89)**
**No primary care provider**	**3.61 (1.43–9.13)**	1.99 (0.66–6.04)	–	1.96 (0.61–6.22)
**Current / ex-smoker (versus never smoker)**	**1.99 (1.14–3.43)**	**1.99 (1.06–3.75)**	–	**2.29 (1.12–4.67)**
***Clinical variables and comorbid conditions***				
**Chronic disability or wheel chair bound**	1.17 (0.70–1.95)	–	1.20 (0.67–2.15)	1.19 (0.59–2.38)
**BMI ≥30 kg/m** ^**2**^	1.14 (0.68–1.89)	–	1.54 (0.86–2.78)	1.94 (1.00–3.76)
**Family history of breast cancer**	1.30 (0.63–2.68)	–	1.44 (0.67–3.12)	1.59 (0.70–3.62)
**Diabetes mellitus**	0.61 (0.36–1.02)	–	**0.49 (0.26–0.90)**	**0.37 (0.19–0.74)**
**Cerebrovascular accident**	**2.18 (1.10–4.39)**	–	**2.73 (1.27–5.89)**	**2.83 (1.21–6.60)**
**Coronary artery disease**	0.98 (0.57–1.68)	–	1.19 (0.59–2.40)	1.15 (0.52–2.56)
**Hypertension**	0.86 (0.43–1.72)	–	0.96 (0.43–2.11)	1.65 (0.63–4.35)
**Hyperlipidemia**	0.78 (0.47–1.31)	–	0.75 (0.39–1.43)	0.85 (0.41–1.75)
**Atrial fibrillation**	0.86 (0.38–1.99)	–	0.78 (0.30–1.99)	0.90 (0.32–2.53)
**Congestive heart failure**	0.91 (0.53–1.56)	–	1.1 (0.52–2.24)	1.31 (0.59–2.93)
**Depression**	1.13 (0.69–1.88)	–	1.01 (0.58–1.77)	1.20 (0.63–2.85)
**Chronic lung disease (COPD/ILD/Asthma)**	0.95 (0.57–1.57)	–	0.78 (0.45–1.34)	0.55 (0.29–1.07)
**Chronic Kidney Disease (CKD)**	0.97 (0.54–1.74)	–	1.1 (0.54–2.27)	1.06 (0.48–2.34)

^**a**^ Adjusted model for social and demographic factors

^**b**^ Adjusted model for clinical variables and comorbid conditions

^**c**^ Adjusted model for both socio-demographic and clinical comorbidities from model 1 and 2.

When both socio-demographic and medical comorbid predictor variables were simultaneously analyzed in the models, the abovementioned variables remained statistically significant with little change in the magnitude of the associations, Table 2.

## Discussion

This study is the first attempt to identify and quantify risk factors associated with non-adherence to breast cancer screening among hospitalized women age 50–74 years. Using multivariable analysis model accounting for both socio-demographic and clinical variables simultaneously, low income, current/ex-smoking status, and a history of stroke were each independently associated with non-adherence to breast cancer screening among hospitalized women. These results are not dissimilar from previous studies looking at other populations that reported screening disparities among women with low socioeconomic status, and low levels of education who fail to engage in screening or preventive testing [8,21–26]. Because hospitalized women have been found to be amenable to breast cancer screening if offered during the hospitalization [11], and even willing to pay out of pocket to offset a part of the cost associated with inpatient mammography [27], the results of this current study may be helpful in identifying subgroups to target with initial inpatient screening interventions.

Although, prior studies have reported that women with disability [28], depression [29], and morbid obesity (BMI > 40 kg/m^2^) [30,31] were less likely to adhere to cancer screening recommendations, these associations with screening mammography were not seen in our cohort of hospitalized women. While others have also shown that women with a family history of breast cancer are more likely to be up to date with their screening [23–25], we also did not find such an association. These results speak to the uniqueness of this study’s population—women who are hospitalized. Truly patient-centered approaches to care involve meeting patients “where they are” philosophically and respecting their preferences. If the literal “where they are” is in the hospital, we might need to evolve in our traditional thinking about the time and place to accomplish screening goals. Patient-centeredness may begin by clearly communicating benefits and harms associated with screening mammography, including breast cancer/all-cause mortality benefits and false-positive rates to ensure informed decision-making [32]. Recent data from US sources estimated that for every 1,000 women undergoing biennial mammography screening over 20 years from age 50, 200 women will have at least one false positive test, 30 will undergo a biopsy, and breast cancer will be over-diagnosed in 15 women [33]. Basic comprehensible language using absolute rates associated with mammography screening should be encouraged as the preferred way to relay the information to patients [34]. It is not known why fewer hospitalized women are up-to-date with their mammograms, but if transportation and remembering to get it done are truly major barriers [11], then seizing the chance to get it done while they are admitted may in fact be the most effective solution.

Several limitations of this study should be considered. First, this study was conducted at a single hospital. Second, while we attempted to evaluate both socio-demographic and health care burden/access factors that can potentially influence patients’ adherence to breast cancer screening, we may not have accounted for all relevant factors. Third, it is unknown whether the pattern of non-adherence with screening remains stable over time. The analyses performed represent data collected at one point time. That said, some women studied have never had a mammogram. Finally, one could argue that implications are only relevant if these non-adherent women would actually agree to have mammograms if they were ordered. In our experience caring for hospitalized patients, few patients refuse tests that are recommended by the physicians caring for them in the hospital.

## Conclusion

There is a need to optimize screening initiatives for breast cancer as early detection translates into reduced mortality. This study has identified the patient-specific factors that are independently associated with being overdue for mammography among hospitalized women; low income, current or ex-smoker, and with history of stroke. While it is not yet common practice for hospital based physicians to extensively review preventive care needs with the patients they are seeing, knowing that these groups are especially likely to be overdue for mammography may incite them to ask, educate, counsel, and even order the test. Such efforts may in turn have a public health impact on the most common cancer affecting women.
